# Interleukin4Rα (IL4Rα) and IL13Rα1 Are Associated with the Progress of Renal Cell Carcinoma through Janus Kinase 2 (JAK2)/Forkhead Box O3 (FOXO3) Pathways

**DOI:** 10.3390/cancers11091394

**Published:** 2019-09-18

**Authors:** Mi-Ae Kang, Jongsung Lee, Sang Hoon Ha, Chang Min Lee, Kyoung Min Kim, Kyu Yun Jang, See-Hyoung Park

**Affiliations:** 1 Department of Biological Science, Gachon University, Seongnam 13120, Korea; makang53@hanmail.net; 2 Department of Integrative Biotechnology, Sungkyunkwan University, Suwon 16419, Korea; bioneer@skku.edu; 3 Division of Biotechnology, Chonbuk National University, Iksan 54596, Korea; hasangpos@gmail.com; 4 Department of Bio and Chemical Engineering, Hongik University, Sejong 30016, Korea; yycc456@naver.com; 5 Department of Pathology, Chonbuk National University Medical School, Chonbuk National University, Jeonju 54896, Korea; kmkim@jbnu.ac.kr; 6 Research Institute of Clinical Medicine, Chonbuk National University, Jeonju 54896, Korea; 7 Biomedical Research Institute of Chonbuk National University Hospital, Chonbuk National University Hospital, Jeonju 54896, Korea

**Keywords:** IL4Rα, IL13Rα1, renal cell carcinoma, JAK2, FOXO3

## Abstract

Specific kinds of interleukin (IL) receptors are known to mediate lymphocyte proliferation and survival. However, recent reports have suggested that the high expression of IL4Rα and IL13Rα1 in tumor tissue might be associated with tumorigenesis in several kinds of tumor. We found that a significant association between mRNA level of IL4Rα or IL13Rα1 and the poor prognosis of renal cell carcinoma (RCC) from the public database (http://www.oncolnc.org/). Then, we evaluated the clinicopathological significance of the immunohistochemical expression of IL4Rα and IL13Rα1 in 199 clear cell RCC (CCRCC) patients. The individual and co-expression patterns of IL4Rα and IL13Rα1 were significantly associated with cancer-specific survival (CSS) and relapse-free survival (RFS) in univariate analysis. Multivariate analysis indicated IL4Rα-positivity and co-expression of IL4Rα and IL13Rα1 as the independent indicators of shorter CSS and RFS of CCRCC patients. For the in vitro evaluation of the oncogenic role of IL4Rα and IL13Rα1 in RCC, we knock-downed IL4Rα or IL13Rα1 and observed that the cell proliferation rate was decreased, and the apoptosis rate was increased in A498 and ACHN cells. Furthermore, we examined the possible role of Janus kinase 2 (JAK2), well-known down-stream tyrosine kinase under the heterodimeric receptor complex of IL4Rα and IL13Rα1. Interestingly, JAK2 interacted with Forkhead box O3 (FOXO3) to cause tyrosine-phosphorylation of FOXO3. Silencing IL4Rα or JAK2 in A498 and ACHN cells reduced the interaction between JAK2 and FOXO3. Moreover, pharmacological inhibition of JAK2 induced the nuclear localization of FOXO3, leading to increase apoptosis and decrease cell proliferation rate in A498 and ACHN cells. Taken together, these results suggest that IL4Rα and IL13Rα1 might be involved in the progression of RCC through JAK2/FOXO3 pathway, and their expression might be used as the novel prognostic factor and therapeutic target for RCC patients.

## 1. Introduction

In 2018, about 65,000 Americans were diagnosed with kidney cancer. Among them, about 15,000 died of this disease [1]. Renal cell carcinoma (RCC) is the most common subtype (about 90%) of kidney cancer. Further, 80% of RCC are classified into clear cell renal cell carcinoma (CCRCC) [2,3]. At the time of initial diagnosis, more than 30% of the patients had metastasis and 20–40% of the patients had systemic metastasis after surgery [4]. Surgical resection is the first and effective treatment option for the early stage kidney cancer. However, there is still a high mortality rate in kidney cancer due to local or remote metastasis of cancer, which is usually highly resistant to the conventional chemotherapy and radiation therapy [5,6]. Thus, it is still needed to understand the detail molecular mechanism of RCC development for efficient care.

Interleukins (IL) are involved in mediating various biological function such as lymphocytes (T and B-cell) activation, proliferation, and differentiation [7,8,9]. Usually, each IL has different activity in the immune regulation process after binding to its receptor, but several kinds of ILs may have the common receptor on the cell membrane. IL4 and IL13 have not structurally nor functionally similar characteristics [10]. Both IL4 and IL13 can exert their function through binding to the IL4 receptor (IL4R). IL4Rα is one subunit of IL4R complex with a specific binding affinity for IL4. There are two types of IL4R complex, depending on the heterodimeric protein subunit components. Type I IL4R is composed of IL4Rα and γc subunit. Type II IL4R is composed of IL4Rα and IL13Rαl subunit. After IL4 binds to IL4Rα, IL13 binds to IL13Rαl to form a heterodimeric complex and combine the signal pathway [10,11,12]. Therefore, type II IL4R complex serves as a functional receptor for both IL4 and IL13 [13]. 

It has been reported that IL4 and IL13-mediated signaling pathways play an important role in tumor biology [14]. For example, IL4R complex is abnormally over-expressed in malignant ovarian, brain, lung, breast, pancreatic, colorectal, bladder, and other epithelial cell type tumors, suggesting that IL4R complex may be involved for tumor progression [14,15,16,17,18,19]. In addition, IL4 and IL13 could act as signaling molecules in the tumor microenvironment. Myeloid-derived suppressor cells (MDSCs) are known to be able to inhibit immune responses in the tumor microenvironment [20]. IL4 and IL13 can activate MDSCs to exert immunosuppression leading to promote tumor growth [21,22]. Furthermore, several studies have reported that single nucleotide polymorphisms (SNPs) in the IL4R gene are closely associated with tumor progression [23,24]. The possibility of treating tumors by blocking IL4Rα to reduce IL4 and IL13 signaling has been examined. Targeting IL4R signaling pathway for treating metastatic cancer has already been approved by the U.S. Food and Drug Administration (FDA) [25]. However, there is no direct study describing the possible oncogenic role of IL4R complex in RCC. 

Janus kinase 2 (JAK2)/Signal transducer and activator of transcription 3 (STAT3) and PI3K/Akt are the main signaling pathways under IL4R complex [25,26,27]. It has been reported that the IL4R complex can promote the progress of lots of tumors by activating STAT6 and Akt. It seems that these pathways are closely related to each one to form a cross talking network. JAK2/STAT3 signaling pathway has been studied for the involvement in the development of lots of tumors [28]. STAT3 is an oncogenic transcriptional factor responsible for anti-apoptotic proteins such as Bcl2 and Bclxl [28]. STAT3 can be tyrosine-phosphorylated by JAK2 and the activated STAT3 translocates from cytoplasm into nucleus to induce the genes related to cell survival and proliferation. According to many recent studies, activated JAK2/STAT3 signaling has been observed in several kinds of tumors such as lung, prostate, and gastric cancers [29,30,31]. Thus, JAK2/STAT3 could serve as a potential target for cancer therapy. However, presently, there is no detailed study elucidating the relationship between IL4R complex and JAK2 in RCC.

Forkhead box O3 (FOXO3) belongs to the forkhead gene family and encodes FOXO3 transcriptional factor responsible for the regulation of genes associated with apoptosis, autophagy, and longevity [32,33,34,35]. FOXO3 is one of the well-known tumor-suppressive transcriptional factors [36]. For instance, in breast and leukemia cancer cells, anti-cancer drugs inhibit tumor development via upregulation of FOXO3 and B cell lymphoma 2 like 11 (Bim) expression [37,38,39]. Moreover, FOXO3 plays a pivotal role in cell cycle arrest through p27 upregulation and can induce G2/M phase cell cycle arrest in breast cancer cells [40,41]. In addition, FOXO3 has been studied for the downregulation of the oncogenic Myc transcriptional factor [42,43,44]. In RCC, the protein expression level of FOXO3 has been reported to be significantly downregulated. However, the mRNA expression level of FOXO3 was not much changed, implying that there might be the post-transcriptional regulation for FOXO3 protein in RCC [45].

In this study, we focus on the clinical outcomes, biological function, and molecular mechanisms of the expression of IL4Rα and IL13Rα1 in RCC progression. Upregulated IL4Rα and IL13Rα1 expression is sufficiently associated with clinical T stage and reduced overall survival of CCRCC patients and down-regulation of IL4Rα and IL13Rα1 expression induced the cell cycle arrest and apoptosis in A498 and ACHN cells. Mechanistically, IL4Rα and IL13Rα1 could increase JAK2 signaling pathway and suppress tumor-suppressive activity of FOXO3. Interestingly, JAK2 interacted with FOXO3 to cause tyrosine-phosphorylation of FOXO3. These results suggest that IL4Rα and IL13Rα1 might be involved in the progression of RCC through JAK2/FOXO3 pathway, and their expression might be used as the novel prognostic factor and promising therapeutic target for RCC patients.

## 2. Results

### 2.1. Immunohistochemical Expression of IL4Rα and IL13Rα1 Are Associated with Poor Prognosis of CCRCC Patients

**Figure 1:**
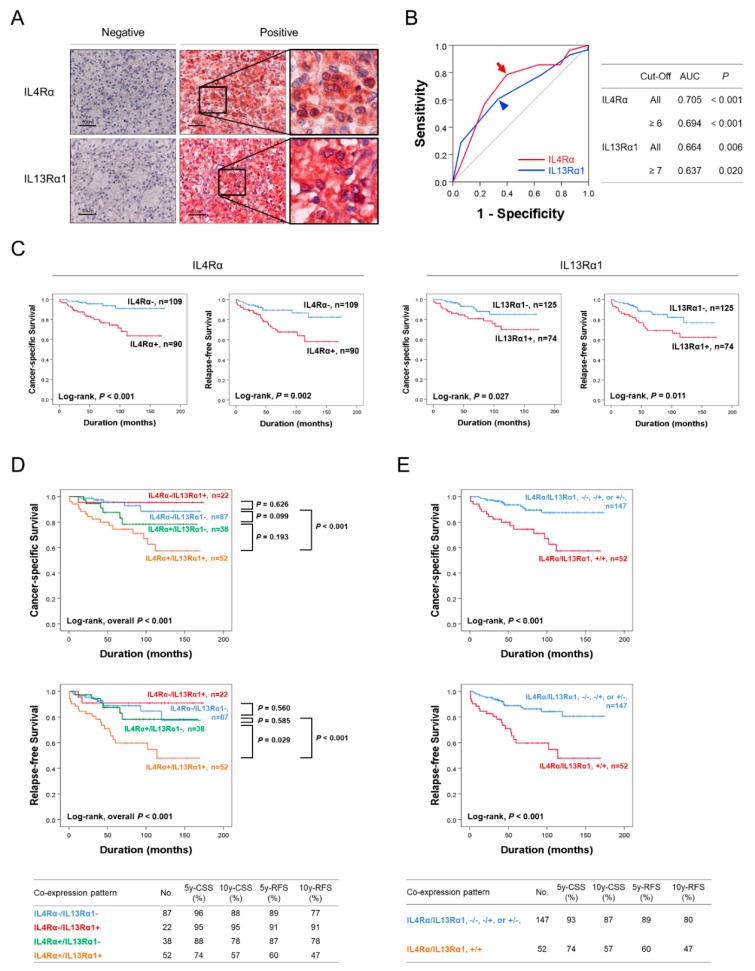
Immunohistochemical expression and survival analysis for the expression of interleukin4 receptor α (IL4Rα) and interleukin13 receptor α1 (IL13Rα1) in clear cell renal cell carcinoma (CCRCC). (**A**) Immunohistochemical expression of IL4Rα and IL13Rα1 in CCRCC tissue. Original magnification, ×400. (**B**) Analysis of sensitivity and specificity of the immunohistochemical staining score of IL4Rα and IL13Rα1 in CCRCC for the event of cancer-specific survival (death of the patient by CCRCC) by receiver operator characteristic curves. The cutoff points were determined at the highest area under the curve (AUC). Red arrow indicates a cutoff point for the IL4Rα immunostaining and blue arrowhead indicates a cut-off point for the IL13Rα1 immunostaining. (**C**) Kaplan-Meier survival analysis for cancer-specific survival and relapse-free survival according to the immunohistochemical positivity for IL4Rα and IL13Rα1 in 199 CCRCC. (**D**) Kaplan-Meier survival analysis in IL4Rα−/IL13Rα1−, IL4Rα−/IL13Rα1+, IL4Rα+/IL13Rα1−, and IL4Rα+/IL13Rα1+ subgroups according to the positivity for IL4Rα and IL13Rα1 expressions. (**E**) Kaplan-Meier survival analysis in two subgroups according to the co-expression patterns of IL4Rα and IL13Rα1; favorable (IL4Rα−/IL13Rα1−, IL4Rα−/IL13Rα1+, or IL4Rα+/IL13Rα1−) and poor prognostic (IL4Rα+/IL13Rα1+) subgroups. 5y-cancer-specific survival (CSS); 5-year cancer-specific survival rate, 10y-CSS; 10-year cancer-specific survival rate; 5y-RFS; five-year relapse-free survival rate, 10y- relapse-free survival (RFS); 10-year relapse-free survival rate.

When we searched OncoLnc public database, higher expression of mRNA of IL4Rα or IL13Rα1 was significantly associated with CCRCC patients (Log-rank, IL4Rα; *p* < 0.001, IL13Rα1; *p* = 0.001) (Figure S1). Similarly, high levels of IL4 and IL13 are detected in the tumor micro-environment, peripheral blood of prostate, bladder, and breast cancer patients. Therefore, the expression of IL4Rα and IL13Rα1 might be used as a new diagnostic and prognostic marker of CCRCC patients. In human CCRCC tissue, the expression of IL4Rα and IL13Rα1 were seen in both the cytoplasm and nuclei of tumor cells (Figure 1A). The cutoff points for immunohistochemical staining scores for IL4Rα and IL13Rα1 expression to classify negative- and positive-subgroups were six and seven, respectively (Figure 1B). At these cutoff points, 45.2% (90 of 199) and 37% (74/125) of CCRCC were subgrouped as IL4Rα-positive and IL13Rα1-positive groups, respectively (Table 1). In addition, there was a significant association between IL4Rα-positivity and IL13Rα1-positivity (*p* < 0.001). The IL13Rα1-positivity was significantly associated with higher tumor stage (*p* = 0.019) (Table 1). The factors significantly associated with both cancer-specific survival (CSS) and relapse-free survival (RFS) in univariate survival analysis, were sex, age of patients, tumor size, tumor stage, lymph node metastasis, and immunohistochemical expressions of IL4Rα and IL13Rα1 (Table 2). The IL4Rα-positivity had a 4.5-fold (95% confidence interval (95% CI); 1.848–11.250, *p* < 0.001) greater risk of death from CCRCC and a 2.8-fold (95% CI; 1.413–5.570, *p* = 0.003) greater risk of relapse or death from CCRCC. The IL13Rα1-positivity showed a 2.3-fold (95% CI; 1.076–4.961, *p* = 0.032) greater risk of death and a 2.2-fold (95% CI; 1.185–4.314, *p* = 0.013) greater risk of relapse or death of CCRCC patients (Table 2). The Kaplan-Meier survival curve for CSS and RFS, according to IL4Rα- and IL13Rα1-positivity are presented in Figure 1C. Furthermore, based on the molecular relationship between IL4Rα and IL13Rα1, we evaluated the clinicopathologic significance of co-expression pattern of IL4Rα and IL13Rα1 in CCRCCs. As shown in Figure 1D, co-expression pattern of IL4Rα and IL13Rα1 was significantly associated with CSS (Log-rank, overall *p* < 0.001) and RFS (Log-rank, overall *p* < 0.001). The 5-year- and 10-year-CSS of IL4Rα-/IL13Rα1- subgroup was 96% and 88%, respectively. The 5-year- and 10-year-CSS of IL4Rα+/IL13Rα1+ subgroup was 74% and 57%, respectively. However, despite the overall prognostic significance of four-subgroups of co-expression patterns of IL4Rα and IL13Rα1, the difference of survival between each subgroup was not significant (Figure 1D). Therefore, based on Kaplan-Meier survival curve for the four-subgroups of co-expression pattern of IL4Rα and IL13Rα1, we re-subgrouped to favorable (IL4Rα−/IL13Rα1−, IL4Rα−/IL13Rα1+, or IL4Rα+/IL13Rα1−) and poor prognostic (IL4Rα+/IL13Rα1+) subgroups (Figure 1E). This subgrouping for the co-expression patterns of IL4Rα and IL13Rα1 was significantly associated with age (*p* = 0.007), tumor size (*p* = 0.029), tumor stage (*p* = 0.027), and lymph node metastasis (*p* = 0.017) (Table 1), and significantly associated with CSS (Log-rank, *p* < 0.001)and RFS (Log-rank, *p* < 0.001) (Figure 1E). Especially, the 5-year- and 10-year-CSS of the favorable prognostic subgroup was 93% and 87%, respectively. In contrast, the 5-year- and 10-year-CSS of the poor prognostic subgroup was 74% and 57%, respectively (Figure 1E). The poor prognostic subgroup showed a 3.7-fold (95% CI; 1.771–7.933, *p* < 0.001) greater risk of death and a 3.4-fold (95% CI; 1.833–6.557, *p* < 0.001) greater risk of relapse or death of CCRCC patients (Table 2). When we performed multivariate analysis with sex, age, tumor stage, histologic nuclear grade, tumor necrosis, and the expression of IL4Rα and IL13Rα1, tumor stage (CSS; *p* < 0.001, RFS; *p* < 0.001) and IL4Rα expression (CSS; *p* = 0.001, RFS; *p* = 0.004) were the independent prognostic indicators for both CSS and RFS (Table 3, model 1). The IL4Rα-positivity had a 4.3-fold (95% CI; 1.753–10.713) greater risk of death and a 2.7-fold (95% CI; 1.372–5.422) greater risk of relapse or death of CCRCC patients (Table 3). When we performed multivariate analysis with inclusion of co-expression pattern of IL4Rα and IL13Rα1 instead of the expression of IL4Rα and IL13Rα1, co-expression pattern of IL4Rα and IL13Rα1 was an independent prognostic indicator for CSS (hazard ratio; 3.286, 95% CI; 1.542–7.000, *p* = 0.002) and RFS (hazard ratio; 3.158, 95% CI; 1.662–6.000, *p* < 0.001) (Table 3, model 2).

### 2.2. Silencing of IL4Rα or IL13Rα1 Decreases Cell Proliferation Rate and Increases Cell Cycle Arrest and Apoptosis in A498 and ACHN Cells

**Figure 2:**
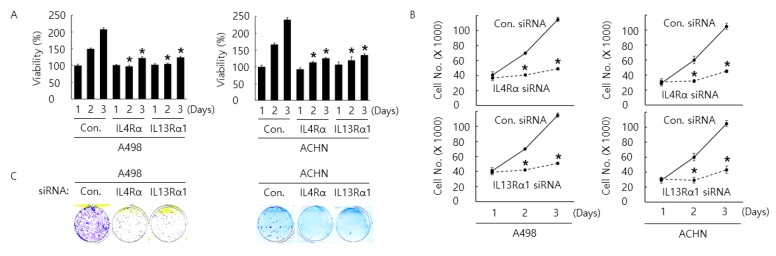

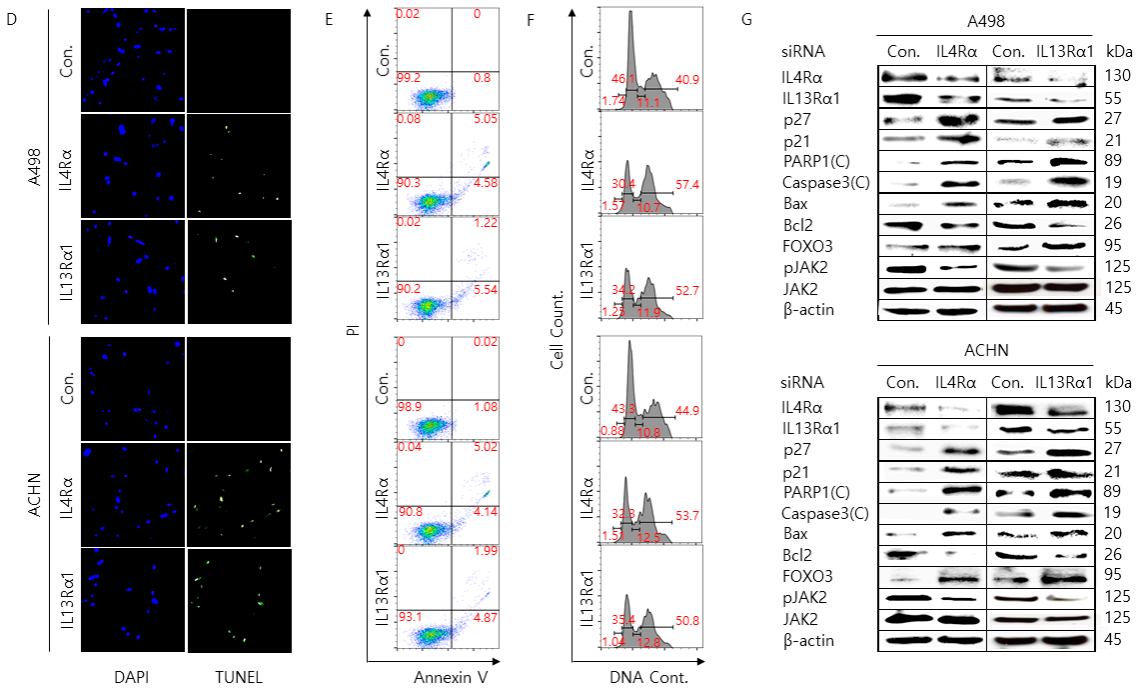
Anti-cancer effect by transfection of siRNA against IL4Rα or IL13Rα1 in A498 and ACHN cells. Time-dependent anti-cancer effect by transfection of siRNA against IL4Rα or IL13Rα1 in A498 and ACHN cells for 24, 48, and 72 h incubation after transfection. Cell viability and proliferation rate was determined by WST-1 assay (**A**) and cell counting assay (**B**), respectively. This result is representative data from three biological replicates, and the error bar indicates standard error (STE). * indicates the *p*-value < 0.05. (**C**) Anti-colony formation ability by transfection of siRNA against IL4Rα or IL13Rα1 in A498 and ACHN cells was determined by colony formation assay for 14 days after transfection. These results are representative data from three biological replicates. Apoptosis in A498 and ACHN cells transfected with siRNA against IL4Rα or IL13Rα1 for 48 h after transfection was determined by terminal deoxynucleotidyl transferase dUTP nick end labeling (TUNEL) assay (**D**) and Annexin V staining analysis (**E**). Cell cycle arrest was determined by cell cycle analysis (**F**). This result is representative data from three biological replicates. (**G**) Western blotting analysis of proteins related to apoptosis and cell cycle arrest in A498 and ACHN cells transfected with siRNA against IL4Rα or IL13Rα1 for 48 h after transfection. β-actin was used for a gel-loading control. Magnification for (**D**): ×20.

In human CCRCC tissue, we found a significant association between the expression IL4Rα and IL13Rα1 and poor prognostic properties. When we examined the protein expression level of IL4Rα in several kinds of RCC cell lines, we observed that ACHN and A498 cells showed relatively the higher protein expression level of IL4Rα than others (Figure S2). Therefore, we evaluated cell proliferation, arrest, and apoptosis after inducing knock-down of IL4Rα or IL13Rα1 in human RCC A498 and ACHN cells. In vitro cell assays (WST-1, cell counting, colony formation, cell cycle analysis, terminal deoxynucleotidyl transferase dUTP nick end labeling (TUNEL), and Annexin V staining assay) showed that knockdown of IL4Rα or IL13Rα1 with siRNA decreased cell proliferation rate and increased cell cycle arrest and apoptosis in A498 and ACHN cells (Figure 2A–F). In addition, western blotting analysis indicated that knockdown of IL4Rα or IL13Rα1 with siRNA increased cleaved poly [ADP-ribose] polymerase 1 (PARP1), cleaved caspase3, Bax, p21, p27, and FOXO3 expression but decreased Bcl2 expression (Figure 2G and Figure S4). Interestingly, knockdown of IL4R complex component caused the downregulation of each other. Then, we examined the phosphorylation level of JAK2 as one of down-stream kinases under the heterodimeric receptor complex of IL4Rα and IL13Rα1. The phosphorylation level of JAK2 was also downregulated by knockdown of IL4Rα or IL13Rα1 with siRNA. Collectively, these results suggest that knockdown of IL4Rα or IL13Rα1 with siRNA transfection is closely involved in proliferation, arrest, and apoptosis in A498 and ACHN cells as well as causing inhibition of JAK2 phosphorylation.

### 2.3. JAK2 Interacts with FOXO3 to Cause Tyrosine-Phosphorylation of FOXO3

**Figure 3:**
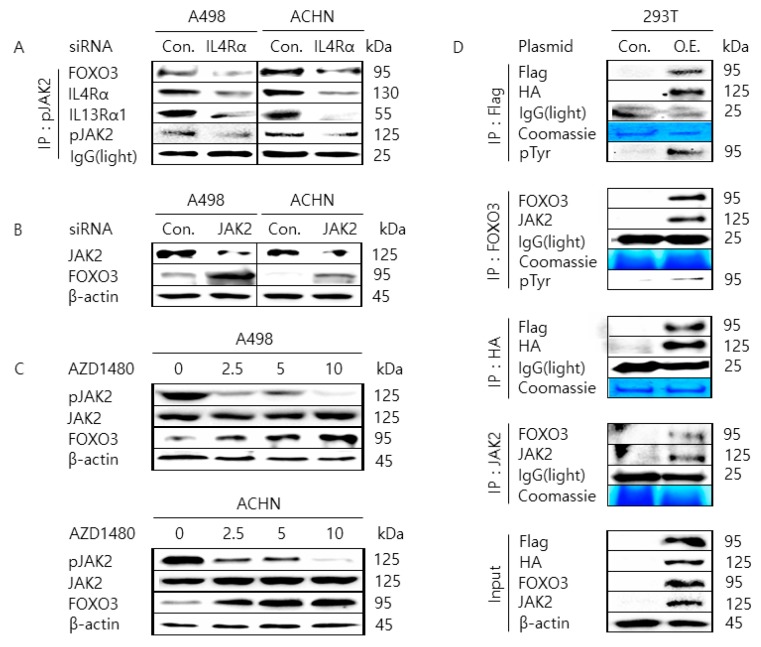
Interaction between Janus kinase 2 (JAK2) and Forkhead box O3 (FOXO3) protein in A498 and ACHN. (**A**) Silencing IL4Rα in A498 and ACHN cells reduced the interaction between JAK2 and FOXO3. Cells were transfected with control siRNA or siRNA against IL4Rα, and then cell lysates were immunoprecipitated with the anti-pJAK2 antibody. The immunoprecipitated proteins were resolved on the sodium dodecyl sulfate polyacrylamide gel electrophoresis (SDS-PAGE) and immunoblotted by anti-IL4Rα, IL13Rα1, FOXO3, and pJAK2 antibody. The light chain of IgG was used for the loading control. (**B**) 293T cells were co-transfected with HA- JAK2 and Flag-FOXO3 (O.E.) or a control plasmid DNA (pCMV3-C-HA and pECE, Con.) as indicated. Then cell lysates were immunoprecipitated with anti-Flag, FOXO3, HA, JAK2, or pTyr antibody. The immunoprecipitated proteins were resolved on the SDS-PAGE and immunoblotted by the indicated antibody, respectively. The light chain of IgG and Coomassie Blue staining of SDS-PAGE were used for the loading control. (**C**) Silencing JAK2 in A498 and ACHN cells increased the expression of FOXO3. Cells were transfected with control siRNA or siRNA against JAK2, and then cell lysates were resolved on the SDS-PAGE and immunoblotted by anti-FOXO3 and JAK2 antibody. β-actin was used for the loading control. (**D**) Pharmacological inhibition of JAK2 in A498 and ACHN cells increased the expression of FOXO3. Cells were treated with dimethyl sulfoxide (DMSO) vehicle control or the indicated concentration of AZD1480, and then cell lysates were resolved on the SDS-PAGE and immunoblotted by anti-FOXO3, pJAK2, and JAK2 antibody. β-actin was used for the loading control.

When we examined the expression of FOXO3 in A498 and ACHN cells transfected with IL4Rα or IL13Rα1 siRNA, we found that the level of FOXO3 appeared to be inversely correlated with the phosphorylation (activation) status of JAK2. Therefore, we tested if JAK2 might interact with FOXO and contribute to a decrease in FOXO3 expression or not. As shown in Figure 3A, we observed that JAK2 protein interacted with FOXO3 protein where the protein interaction was weakened by the transfection of IL4Rα siRNA in A498 and ACHN cells. Then, we found that the expression level of FOXO3 was increased by the transfection of JAK2 siRNA or the treatment of AZD1480, one of the pharmacological inhibitors of JAK2 kinase, in A498 and ACHN cells (Figure 3B,C). To confirm whether JAK2 interacts with FOXO3, we performed co-immunoprecipitation with an antibody against Flag or FOXO3 followed by immunoblotting analysis with an antibody against HA or JAK2 in 293T cell co-transfected with over-expressing plasmid DNAs (pECE Flag-FOXO3 and pCMV3-C-HA-JAK2) (Figure 3D). Moreover, we performed a reciprocal co-immunoprecipitation with an antibody against HA or JAK2 followed by immunoblotting analysis with an antibody against an antibody against Flag or FOXO3. As shown in Figure 3D, protein interaction between JAK2 and FOXO3 was increased in 293T cell co-transfected with over-expressing plasmid DNAs (pECE Flag-FOXO3 and pCMV3-C-HA-JAK2) compared to 293T cell transfected with the control vectors. Also, we could observe that the tyrosine-phosphorylation level of FOXO3 increased in 293T cell co-transfected. These results implicate that JAK2 interacts with FOXO3 to cause tyrosine-phosphorylation of FOXO3 and regulate the protein level of FOXO3.

### 2.4. Pharmacological Inhibition of JAK2 Induces the Nuclear Localization of FOXO3 and Increases FOXO3 Protein Stability

**Figure 4:**
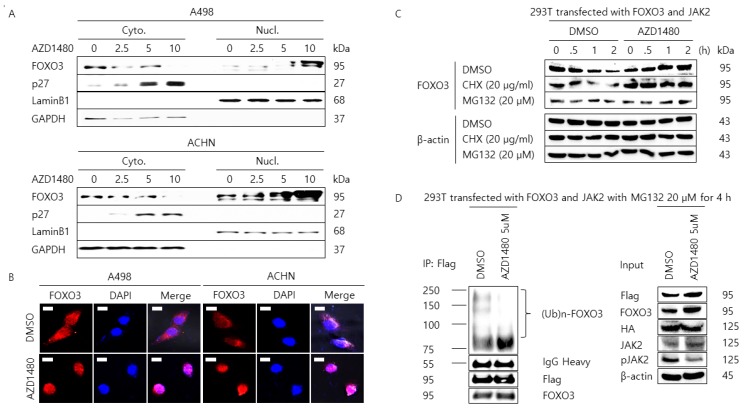
The nuclear localization of FOXO3 by inhibition of JAK2. (**A**) Cells were treated with DMSO vehicle control or the indicated concentration of AZD1480 (0, 2.5, 5, and 10 μM) for 48 h, washed with PBS, trypsinized, and lysed in the cytoplasmic fractional buffer. After separating the cytoplasmic fraction, the remaining pellet was washed with washing buffer and lysed with nuclear fractional buffer, and the supernatant was collected for the nuclear fraction. Glyceraldehyde 3-phosphate dehydrogenase (GAPDH) and Lamin B1 were used for a gel loading control for the cytoplasmic and nuclear protein fractions, respectively. (**B**) Pharmacological inhibition of JAK2 induces the nuclear localization of FOXO3 in A498 and ACHN. Cells were treated with DMSO vehicle control or AZD1480 (5 μM) for 2 h and then incubated with a primary antibody against FOXO3 followed by Alexa 594-conjugated anti-mouse secondary antibody. The nuclei were counterstained by 4′,6-diamidino-2-phenylindole (DAPI). Fluorescence images were captured with a confocal microscope. Scale bar: 5 μm (**C**) Pharmacological inhibition of JAK2 increases the protein stability of FOXO3 in 293T cells transfected with pECE Flag-FOXO3 and pCMV3-C-HA- JAK2 plasmid DNA via a proteasome-mediated pathway. Next, 293T cells were treated with DMSO (control vehicle), cycloheximide (CHX, 20 μg/mL), or MG-132 (20 μM) with/without AZD1480 (5 μM) for the indicated time and then cell lysates were resolved on the SDS-PAGE and immunoblotted by the anti-FOXO3 antibody. β-actin was used for the loading control. (**D**) Pharmacological inhibition of JAK2 decreases the ubiquitination of FOXO3 in 293T cells transfected with pECE Flag-FOXO3 and pCMV3-C-HA-JAK2 plasmid DNA. 293T cells were treated with DMSO (control vehicle) or MG132 (20 μM) with/without AZD1480 (5 μM) for the indicated time and then cell lysates were immunoprecipitated with anti-FOXO antibody and blotted with anti-ubiquitin, Flag, and FOXO3 antibody. The heavy chain of IgG was used for the loading control. For input analysis, cell lysates were resolved on the SDS-PAGE and immunoblotted by anti- JAK2, pJAK2, and FOXO3 antibody. β-actin was used for the loading control.

The half-life of FOXO3 protein is controlled by the proteasomal degradation after its phosphorylation [46,47,48]. Since we could find that FOXO3 protein interacts with JAK2 protein and the tyrosine-phosphorylation of FOXO3 was induced by JAK2, we tried to determine whether JAK2 affects the FOXO3 protein location, level, and ubiquitination status. As shown in Figure 4A, the cytoplasmic level of FOXO3 protein was decreased by AZD1480 treatment in A498 and ACHN cells, while the nuclear level of FOXO3 protein was significantly increased with a dose-dependent manner. Further, the cytoplasmic level of p27 protein was increased by AZD1480 treatment in a dose-dependent manner. These data were confirmed by the confocal analysis for staining FOXO3 protein in A498 and ACHN cells treated with AZD1480, which showed the nuclear accumulation of FOXO3 after treatment of AZD1480 (Figure 4B). These results suggest that AZD1480 could trigger the translocation of the FOXO3 protein from the cytoplasm into the nucleus in A498 and ACHN cells. Then, we found that the expression level of FOXO3 was decreased within 2 h in 293T cells co-transfected with over-expressing plasmid DNAs (pECE Flag-FOXO3 and pCMV3-C-HA-JAK2) but the expression level of FOXO3 was increased up to 4 h in the same 293T cells treated with AZD1480 with/out the treatment of CHX and MG132 (Figure 4C) suggesting that the proteasomal degradation of FOXO3 is controlled by AZD1480 treatment. Moreover, as shown in Figure 4D, immunoprecipitation analysis result with antibody against Flag after incubation of MG132 showed that poly-ubiquitination of FOXO3 was considerably weakened in 293T cells treated with AZD1480 compared to dimethyl sulfoxide (DMSO) vehicle control which is consistent with the above experimental results about the proteasomal degradation of FOXO3.

### 2.5. Pharmacological Inhibition of JAK2 Decreases Cell Proliferation Rate and Increases Cell Cycle Arrest and Apoptosis in A498 and ACHN Cells

**Figure 5:**
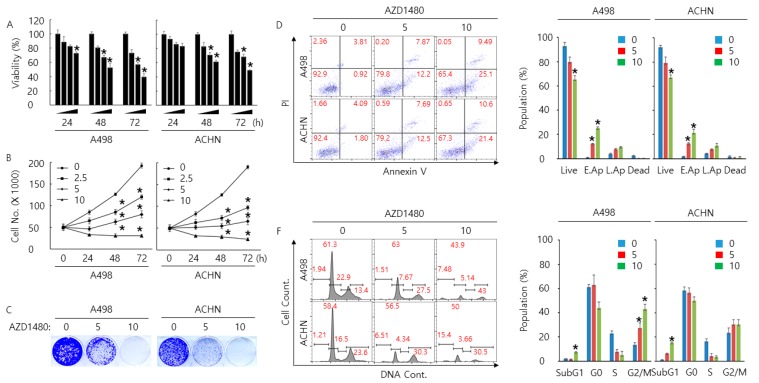

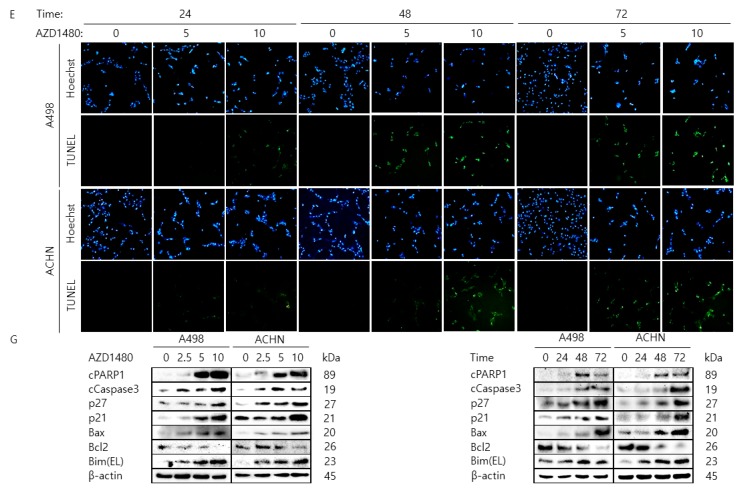
Anti-cancer effect by AZD1480 treatment in A498 and ACHN cells. (**A**) Dose and time-dependent anti-cancer effect by the indicated concentration of AZD1480 treatment in A498 and ACHN cells for 24, 48, and 72 h. Cell viability and proliferation rate was determined by WST-1 assay (**A**) and cell counting assay (**B**), respectively. This result is representative data from three biological replicates, and the error bar indicates standard error (STE). * indicates the *p*-value < 0.05. (**C**) Anti-colony formation ability by the indicated concentration of AZD1480 treatment in A498 and ACHN cells was determined by colony formation assay for 14 days. This result is representative data from three biological replicates. Apoptosis in A498 and ACHN cells treated by the indicated concentration of AZD1480 was determined by Annexin V staining analysis (**D**) and TUNEL assay (**E**). Live: live cells, E.Ap: early apoptotic cells, L.Ap: late apoptotic cells, and dead: dead cells. Cell cycle arrest was determined by cell cycle analysis (**F**). These results are representative data from three biological replicates. (**G**) Dose and time-dependent western blotting analysis of proteins related to apoptosis and cell cycle arrest in A498 and ACHN cells treated by the indicated concentration of AZD1480. β-actin was used for a gel-loading control. Magnification for (**E**): ×20.

To determine the anti-cancer effect of AZD1480 treatment, we performed cell proliferation, arrest, and apoptosis assays in A498 and ACHN cells treated with AZD1480. In vitro cell assays (WST-1, cell counting, colony formation, cell cycle analysis, TUNEL, and Annexin V staining assay) showed that AZD1480 treatment decreased cell proliferation rate and increased cell cycle arrest and apoptosis in A498 and ACHN cells with dose and time-dependent manner (Figure 5A–F). Furthermore, knockdown of JAK2 with siRNA also decreased cell proliferation rate in A498 and ACHN cells (Figure S3A–C). Western blotting analysis indicated that AZD1480 treatment significantly increased cleaved PARP1, cleaved caspase3, Bax, p21, p27, Bim(EL) and FOXO3 expression but decreased Bcl2 expression with dose and time-dependent manner (Figure 5G). Collectively, these results suggest that pharmacological inhibition by AZD1480 treatment is closely involved in proliferation, arrest, and apoptosis in A498 and ACHN cells through FOXO3 activation.

## 3. Discussion

IL4 and IL13 are closely linked and expressed mono-allelically in Th2 cells [49]. IL4 and IL13 have many biological functions. One of them is to stimulate B and T-cell proliferation and differentiation of B-cell into plasma cell [50]. IL4 and IL13 share various biological functions [51]. They regulate immune responses under normal physiological conditions as well as in cancer [14]. IL4 and IL13 also play crucial roles in tumor biology and tumor immunology via activation of immune cells in the tumor microenvironment [14]. For example, it is reported that IL4 promoted tumor development via p21 mediated activation of STAT6 signaling pathways in IL4 downregulated melanoma models [52]. IL4 and IL13 bind to their receptors specifically, and both cytokines can have effects on cancer cells expressing appropriate receptors [53,54]. The receptor is made up of the heterodimer, IL4Rα, and IL13Rα1 chain [55]. IL4 and IL13 bind to IL4Rα and IL13Rα1 chains, forming functional structures in cancer cells [14]. It is reported that IL4Rα is overexpressed in human breast cancer and silencing of IL4Rα attenuated growth of metastatic breast cancer cells [56]. Moreover, IL4Ra expression activates colon tumor growth [57]. IL4Ra has also been recognized as a risk factor of pancreatic cancer [58]. IL4Rα activates directly mammary tumor metastasis [59]. IL4 and IL4Ra are related to the colorectal adenoma-carcinoma proliferation and the capacity of metastasis [24]. IL13Rα1 is an important to target of cancer therapy in human head and neck cancer animal models [60]. Overexpressed IL13Rα1 in tumor cells is closely related to patients with breast cancer [18]. Therefore, these receptors have been recognized as the main target of cancer treatment [61]. However, so far, there is no detailed research on the oncogenic role of IL4Rα and IL13Rα1 in RCC. 

In this study, immunohistochemical expression of IL4Rα and IL13Rα1 was significantly associated with shorter CSS and RFS of CCRCC patients. Moreover, individual expression of IL4Rα and co-expression pattern of IL4Rα and IL13Rα1 were independent poor prognostic indicators of CCRCC patients by multivariate analysis. Especially, IL4Rα^+^IL13Rα1^+^ subgroup of CCRCC significantly associated with larger tumor size, higher tumor stage, and lymph node metastasis, and predicted a 3.2-fold greater risk of death of CCRCC patients compared with IL4Rα^−^IL13Rα1^−^/IL4Rα^−^IL13Rα1^+^/IL4Rα^+^IL13Rα1^−^ subgroups (Table 1 and Table 3). These results suggest that the expression of IL4Rα and IL13Rα1 and their co-operative expression patterns are important in the progression of CCRCCs. As shown in Figure 1A, IL4Rα and IL13Rαl were detected in the cytoplasm and nuclei in CCRCC tissue samples. IL4Rα or IL13Rαl proteins are located in cell-membrane and composed of outer, inner, and cell membrane-integrated part. Outer part of IL4Rα or IL13Rαl protein recognizes IL4 or IL13. Inner part of IL4Rα or IL13Rαl protein binds to and activates the down-stream signaling proteins such as JAK2. Thus, we thought that not only the membrane expression but also the cytoplasmic expression of IL4Rα or IL13Rαl proteins might be detected by immunohistochemical staining analysis. Furthermore, the Human Protein Atlas public database (http://www.proteinatlas.org) indicates that both of IL4Rα and IL13Rα1 are expressed in the cytoplasmic membrane and nuclear of cells and the images from the Human Protein Atlas public database showed the cytoplasmic and nuclear expression of IL4Rα and IL13Rα1 as below captured. As shown in Figure 1D, IL4Rα−/IL13Rα1+ subgroup seems to lead to better prognosis than IL4Rα−/IL13Rα1− subgroup in terms of both of CSS and RFS rate. Type II IL4R is composed of IL4Rα and IL13Rαl subunit. Mechanistically, IL4 binds to IL4Rα and then IL13 binds to IL13Rαl to form a heterodimeric complex and combine the signal pathway [10,11,12]. Thus, we though that the reverse binding order (IL13 binds to IL13Rαl and then IL4 binds to IL4Rα) might cause abrogate the functional complex forming of type II IL4R, which lead to inhibit RCC development and prolong the survival rate of RCC patients. 

Recent reports showed that IL4Rα and IL13Rα1 are overexpressed and activated in various types of epithelial tumor such as malignant glioma, ovarian, lung, pancreas, and colon carcinoma [17,62]. Therefore, IL4Rα and IL13Rα1 might be an effective therapeutic target of various human malignant tumors. Suppression of IL4 and IL13 as well as IL4Rα and IL13Rα1 might be tumor-suppressive especially in poor prognostic group of cancers expressing both IL4Rα and IL13Rα1 as like IL4Rα^+^IL13Rα1^+^ subgroup of CCRCC. RNA aptamer-mediated inhibition of IL4Rα induces apoptosis of MDSC and tumor-associated macrophage (TAM) which is associated with the tumoral immune escape [63]. Joshi et al. showed the higher expression of IL4Rα in anaplastic thyroid cancer (ATC), a highly aggressive thyroid cancer type than other types of thyroid cancer tissue from patients. They designed the IL4-Pseudomonas exotoxin (IL4-PE) conjugate and the treatment of IL4-PE decreased the colony formation ability of thyroid cancer cells and tumor growth in thyroid xenograft tumor model [64]. Furthermore, IL4R plays an essential role in regulating hepatocellular carcinoma (HCC) cell survival, proliferation, and metastasis and regulates the activation of JAK1/STAT6 and Jnk/Erk1/2 pathways [65]. As shown in the Figure 2G, interestingly, the knockdown of the IL4R complex component caused the downregulation of each other. Type II IL4R is composed of IL4Rα and IL13Rαl subunit. We thought that if one of IL4R complex component is ablated by siRNA transfection, this might cause the protein degradation of each components to be accelerated since each component need to be physically interacted to form the functional complex of type II IL4R. We are planning to investigate if the half-life of IL4Rα or IL13Rαl protein is controlled by the proteasomal degradation after transfection of siRNA against IL4Rα or IL13Rαl into ACHN and A498 cells. In this study, knockdown of IL4R prohibited proliferation and induced apoptosis in HCC cells. Vadevoo et al. reported that they synthesized an IL4R-targeting peptide (IL4RPep-1-K) conjugated with the proapoptotic peptide (KLAKLAK)2 and demonstrated this peptide exerted the selective anti-cancer effect against IL4R-expressing tumor in vitro and in vivo (4T1 breast tumor-bearing mice model) [11]. In fact, our laboratory has been developing the novel drug delivery system using ultrasound and microbubble-liposome complex with IL4RPep-1-K to detect and treat IL4R-expressing tumor [66].

JAKs (JAK1, JAK2, JAK3, and Tyk) has seven domains called Janus homology domains (JH). Among these seven JHs, JH1 is the kinase domain responsible for a tyrosine kinase activity of JAKs and has the conserved tyrosine residues required for JAK activation (e.g., Y1007/Y1008 in JAK2). Phosphorylation of these dual tyrosines leads to the conformational changes in the JAK protein to facilitate the binding of the substrate [67]. JAK2 is a member of JAKs and a non-receptor tyrosine kinase. Compared with other JAKs, JAK2 lacks Src homology binding domains (SH2/SH3) [68]. JAK has been recognized as an important factor because it is closely related to various kinds of diseases [69,70,71]. Therefore, JAK inhibitor may play a crucial role in the treatment of diseases clinically. For instance, selective JAK2 inhibitor TG101348 attenuated myelofibrosis in a murine model of disease [69]. Furthermore, it was reported that JAK inhibitor had provided benefit to myeloproliferative neoplasms patients because it might reduce the production of pro-inflammatory cytokines [72]. FDA has approved several JAK inhibitors, such as Ruxolitinib, Tofacitinib, and Fedratinib [73,74]. Ruxolitinib, a small-molecule inhibitor of JAK1/2, was the first FDA approved drug for the treatment of myelofibrosis [75]. Recently, FDA approved Fedratinib, a small-molecule inhibitor of JAK2, for the treatment of myelofibrosis [76]. 

JAK2/STAT3 signaling pathway is known to be involved in a number of inflammatory, anti-inflammatory signaling pathways, and various pathological regulation processes [77]. It is reported that the expression of IL13Rα1 induced STAT3 activation by IL4 and IL13 in the stimulated human B cells [55]. JAK2/STAT3 signaling pathway plays a crucial role in the growth of breast cancer cells [78]. Inhibition of JAK2/STAT3 signaling pathway induces apoptosis in ovarian cancer cells [79,80]. Furthermore, inhibition of JAK2/STAT3 pathway suppresses the growth of gastric cancer in vitro and in vivo [81]. For treating RCC, JAK2/STAT3 has been recognized as an important target. A recent study showed that activation of JAK2/STAT3 signaling pathway promotes cell proliferation in CCRCC [82]. According to Xin et al, treatment of 786-O human renal cancer cells with AZD1480 showed only limited decrease in cell viability. They used 0, 0.5, and 1 μM of AZD1480 to perform MTS cell viability assay [83]. However, we used 0, 2.5, 5, and 10 μM of AZD1480 to perform WST-1 assay and we could observe the dose-and time dependent cell toxicity effect of AZD1480 in A498 and ACHN cells. When we tested the lower dose of AZD1480 (0.5 and 1 μM) for the cell viability assay, we also found the limited reduction of cell viability in A498 and ACHN cell. Thus, we thought that the range of AZD1480 dose might cause the different anti-cancer effect on the renal cancer cells. Moreover, Icaritin, a kind of flavonoid isolated from *Herba Epimedii*, suppressed JAK2/STAT3 signaling pathway, and proliferation of RCC [84]. Thus, it is important to find an effective reagent to inhibit JAK2/STAT3 signaling pathway in RCC. As described in the results section, we demonstrated that AZD1489, a kind of JAK2 inhibitor, decreased cell proliferation rate and increases cell cycle arrest and apoptosis in A498 and ACHN cells with dose and time-dependent manner. For the expansion of our research, now we are planning to develop a novel JAK2 inhibitor by screening FDA approved drug library to treat RCC.

Over the past decade, the relationship between FOXO3 and cancer cell development has been investigated. Down-regulation of FOXO3 is detected frequently in cancer development [85,86]. The relationship between FOXO3 and RCC also has been studied. Down-regulation of FOXO3 promotes tumor metastasis in RCC 786O cells [45]. Furthermore, FOXO3 upregulates the expression level of miR-30d, a kind of tumor-suppressive microRNAs, transcriptionally in RCC cell lines [87]. FOXO3 is one of the human FOXO transcription factors and know to be inhibited by the phosphorylation-dependent protein degradation in the cytoplasm [48,88]. A recent study demonstrated that Src kinases are required for the regulation of *Drosophila* FOXO3 (dFOXO) [89]. According to them, the introduction of Src42A, one of the constitutively active alleles, into the larval inhibited the starvation-mediated nuclear localization of dFOXO, and pharmacological inhibition of Src activity promoted the nuclear accumulation of dFOXO under the even high nutrient conditions. Src is one of the effector tyrosine kinase under the insulin or insulin-like growth factor (IGF) receptor signaling pathway. Thus far, the regulation of FOXO3 mediated by tyrosine phosphorylation has not been well-investigated. In this study, we also tried to show that JAK2 tyrosine kinase could phosphorylate FOXO3 and pharmacological inhibition of JAK2 induces the nuclear accumulation of FOXO3 in RCC cell lines. We also determined if tyrosine phosphorylation of FOXO3 by JAK2 affects the ubiquitination status of FOXO3. Ubiquitination pattern of FOXO3 protein was decreased significantly when FOXO3 and JAK2 were co-transfected in 293T cells with AZD1480, a kind of JAK2 inhibitor suggesting that ubiquitination of FOXO3 depends on inhibition of JAK2 activity. However, we still have the question which site of tyrosine in FOXO3 could be phosphorylated by JAK2 kinase and which E3 ligase is involved in this specific ubiquitination process. We are currently constructing several tyrosine mutants of FOXO3 to examine which tyrosine sites are phosphorylated by JAK2. Since JAK2/STAT3 and PI3K/Akt are the two kinds of main signaling pathways under IL4R complex, we might think about which signaling pathway plays more important in regulating the protein stability and ubiquitination status of FOXO3 among JAK2/STAT3 and PI3K/Akt. To examine this issue, we are seeking to perform the experiments for analyzing the protein stability and ubiquitination status of FOXO3 in the cells treated with JAK2 or Akt inhibitor as well as knock-downed or knock-outed JAK2 or Akt.

There have been a few reports describing the relationship between JAK2 and FOXO3. Ahn et al. demonstrated that enhanced reactive oxygen species (ROS) level and aberrant PI3K signaling decreased the nuclear localization of FOXO3 and catalase expression in JAK2V617F mutant positive cells. In addition, they showed that JAK2V617F-positive erythroblasts derived from myeloproliferative neoplasm patients also displayed the increased ROS level and reduced nuclear FOXO3 compared with the control erythroblasts [90]. Since JAK2 is known to phosphorylate and activate STATs in various kinds of cells, we might think about the possibility of the protein interaction between FOXO3 and STATs. Ma et al. reported that FOXO3 or FOXO1 binds to STAT3, leading to negatively regulate specificity protein 1 (SP1)-pro-opiomelanocortin (POMC) promoter complex [91]. In addition, Oh et al. showed that FOXO3 or FOXO1 interacts with STAT3 in the cytoplasm to regulates translocalization of FOXO3 or FOXO1 in CD4(+) T cells leading to control the inflammatory responses [92]. As described in the results section, we successfully showed that JAK2 interacts with FOXO3 to cause tyrosine-phosphorylation of FOXO3. Thus, we should consider what is the detailed relationship between JAK2, STAT3, and FOXO3 in cancer cells. For elucidating this issue, we are trying to investigate which phosphorylation step plays more important in RCC development by performing the protein binding experiments among these proteins.

## 4. Materials and Methods

### 4.1. Clear Cell Renal Cell Carcinoma Patients and Tissue Samples

The patients who performed the operation for the clear cell renal cell carcinoma (CCRCC) between July 1998 and August 2011 were evaluated in this study. Original histologic slides, tissue blocks, and medical records were available in 199 cases of CCRCC patients [93]. Clinical information was obtained by reviewing medical records. The histopathologic factors were reevaluated with original histologic slides according to the World Health Organization classification of the renal tumor [94]. Tumor stage was reevaluated according to the eighth edition of the staging system of the American Joint Committee on Cancer [95]. This study obtained institutional review board approval from Chonbuk National University Hospital (IRB No., CUH 2014-05-039-002) and was performed according to the Declaration of Helsinki. The approval contained a waiver for written informed consent based on the retrospective and anonymous character of this study.

### 4.2. Immunohistochemical Staining and Scoring

Immunohistochemical staining in CCRCC tissue sample for IL4Rα and IL13Rα1 were performed in histologic slides from tissue microarrays with one 3.0 mm cores per case. The tissue microarray core established from the tumor components with the highest histologic grade without any degeneration or necrosis. The paraffin-embedded histologic sections derived from the tissue microarray were deparaffinized, and antigen retrieval was performed by boiling in the microwave oven for 20 min in pH 6.0 antigen retrieval solution (DAKO, Glostrup, Denmark). Anti-IL4Rα (1:100, sc-165974, Santa Cruz Biotechnology, Santa Cruz, CA, USA) and anti-IL13Rα1 (1:100, sc-25849, Santa Cruz Biotechnology) antibodies were used as primary antibodies and visualized with the enzyme substrate 3-amino-9-ethylcarbazole. Scoring for the immunohistochemical staining slides was performed by two pathologists (Kyu Yun Jang and Kyoung Min Kim) with consensus by simultaneously observing in multi-viewing microscope without clinicopathologic information. The immunohistochemical staining score obtained by adding staining intensity point and staining area point. The staining intensity pointed from zero to three (0; no staining, 1; weak, 2; intermediate, 3; strong) and staining area pointed from zero to five (0; no staining, 1; 1%, 2; 2–10%, 3: 11–33%, 4; 34–66%, 5; 67–100%) [93,96,97]. Therefore, the score ranged from zero to eight.

### 4.3. Cell Culture

A498, ACHN, and 293T cells (ATCC, Manassas, VA, USA) were maintained in Dulbecco’s modified Eagle’s media (DMEM, Gibco, Waltham, MA, USA) media with 10% fetal bovine serum (FBS, Gibco, Waltham, MA, USA) and 1% streptomycin/penicillin at 37 °C in a humidified incubator containing 5% CO_2_ in the air. Both cell lines were used at passages 4–10 for all experiments.

### 4.4. Chemical Reagents, Antibodies, and Plasmid DNAs

Mouse anti-β-actin, mouse anti-Flag, and mouse anti-HA antibody and the following chemicals and solvents (MG132, cycloheximide, dimethyl sulfoxide (DMSO), glycerol, glycine, sodium chloride, Trizma base, and Tween20) were from Sigma (St. Louis, MO, USA). AZD1480 was from Selleckchem (Houston, TX, USA). Rabbit anti-IL4Rα, rabbit anti-IL13Rα1, mouse anti-PARP1, rabbit anti-FOXO3, mouse anti-Lamin B1, and mouse anti-GAPDH antibodies were from Santa Cruz Biotechnology. Rabbit anti-JAK2, rabbit anti-pJAK2, rabbit anti-Tyr, rabbit anti-cleaved PARP1, rabbit anti-cleaved Caspase3, rabbit anti-Bax, rabbit anti-Bim, rabbit anti-Bcl2, rabbit anti-p21, and rabbit anti-p27 antibodies were from Cell Signaling (Danvers, MA, USA). Goat anti-rabbit (111-035-003) and goat anti-mouse (115-035-003) horseradish peroxidase-conjugated IgG were obtained from Jackson ImmunoResearch (West Grove, PA, USA). Enhanced chemiluminescence (ECL) reagents were obtained from Genedepot (Barker, TX, USA). pECE empty/Flag-FOXO3 and pCMV3-C-HA empty/HA-JAK2 plasmid DNA were from Addgene (Watertown, MA, USA) and Sino Biological (Wayne, PA, USA), respectively.

### 4.5. Water Soluble Tetrazolium Salts 1 (WST-1) Assay

Cells (1 × 10^3^) were seeded in each well of a 96-well plate and incubated for 18 h at 37 °C in a humidified incubator containing 5% CO_2_ in the air. After incubation, cells were treated with DMSO (0.1%) as a control vehicle and the indicated treatment for 24, 48, or 72 h. After incubation, 20 µL of WST-1 solution was added to each well for 4 h. Then, the visible absorbance at 460 nm of each well was quantified using a microplate reader.

### 4.6. Colony Formation Assay

Cells (5 × 10^2^) were seeded in 6-cm dishes and incubated for 18 h at 37 °C in a humidified incubator containing 5% CO_2_ in the air. After incubation, cells were treated with DMSO (0.1%) as a control vehicle or the indicated treatment for 14 days. The colonies were washed twice with PBS, fixed with 3.7% paraformaldehyde, and stained with 1% crystal violet solution in distilled water.

### 4.7. Western Blotting Analysis

Cells were washed with PBS and lysed in lysis buffer (50 mM Tris-HCl, 150 mM NaCl, 2 mM EDTA, 1% Triton X-100, 0.1% SDS, pH 8.0) with protease and phosphatase inhibitors. Cell lysates were centrifuged (10,000× *g*, 4 °C, 10 min), and the supernatants were separated on 10% SDS-PAGE gels and blotted onto nitrocellulose membranes (Bio-Rad Laboratories, Hercules, CA, USA). The membranes were blocked in 3% non-fat dry milk for 1 h at room temperature and probed with the appropriate antibodies. Membranes were then probed with HRP-tagged anti-mouse or anti-rabbit IgG antibodies diluted 1:5,000–1:20,000 in 3% non-fat dry milk for 1 h at room temperature. Chemiluminescence was detected using enhanced chemiluminescence (ECL).

### 4.8. Cytoplasmic and Nuclear Protein Fractionation

Cells were washed with PBS and lysed in cytoplasmic fractional buffer (10 mM HEPES, pH 8.0, 50 mM NaCl, 500 mM sucrose, 1 mM EDTA, 0.5 mM spermidine, 0.15 mM spermine, 0.2% Triton X-100, 1 mM DTT, 2 µM PMSF and 0.15 U/mL aprotinin) at 4 °C for 30 min. Cell lysates were centrifuged (10,000× *g*, 4 °C, 30 min), and the supernatant was collected for the cytoplasmic fraction. The pellet was washed twice the washing buffer (10 mM HEPES pH 8.0, 50 mM NaCl, 25% glycerol, 0.1 mM EDTA, 0.5 mM spermidine and 0.15 mM spermine) and lysed with nuclear fractional buffer (10 mM HEPES pH 8, 350 mM NaCl, 25% glycerol, 0.1 mM EDTA, 0.5 mM spermidine and 0.15 mM spermine) at 4 °C for 30 min. Lysates were centrifuged (10,000× *g*, 4 °C, 30 min), and the supernatant was collected for the nuclear fraction.

### 4.9. Immunofluorescence Analysis

Cells (5 × 10^5^) were seeded in 6-cm dishes and incubated for 18 h at 37 °C in a humidified incubator containing 5% CO_2_ in the air. After incubation, cells were treated with DMSO (0.1%) as a control vehicle and the indicated concentration of AZD1480 for 2 h. Cells were fixed with 4% paraformaldehyde solution, permeabilized with Triton X-100 (0.2%), blocked with bovine serum albumin (BSA, Gibco, Waltham, MA, USA) and incubated with a primary antibody against FOXO3 followed by Alexa 594-conjugated anti-mouse secondary antibody (Invitrogen, Carlsbad, CA USA). After counterstaining with DAPI, fluorescence images were captured with a confocal microscope (Zeiss LSM510 microscope, Carl Zeiss, Jena, Germany). 

### 4.10. Immunoprecipitation Analysis

Cells were washed with PBS and lysed in lysis buffer (50 mM Tris-HCl, 150 mM NaCl, 2 mM EDTA, 1% Triton X-100, 0.1% SDS, pH 8.0) with protease and phosphatase inhibitors. Cell lysates were centrifuged (10,000× *g*, 4 °C, 10 min) and the supernatants were incubated with an antibody by rotating at 4 °C overnight followed by the addition of 20 μL of 50% protein A or G-agarose slurry and rotating for 1 h. Protein A or G-agaroses were collected and washed with lysis buffer. Immunoprecipitants were resolved by 10 or 12% SDS-PAGE and analyzed by Western blotting analysis.

### 4.11. TUNEL Assay

Cells (5 × 10^5^) were seeded in 6-cm dishes and incubated for 18 h at 37 °C in a humidified incubator containing 5% CO_2_ in the air. After incubation, cells were treated with DMSO (0.1%) as a control vehicle (or control siRNA) and the indicated treatment (or siRNA against IL4Rα or IL13Rα1) for 24, 48, or 72 h (or for 48 h). Cells were fixed with 4% paraformaldehyde solution and permeabilized with Triton X-100 (0.2%). Apoptosis was determined by enzymatic labeling of DNA strand breaks with a TUNEL assay kit (DeadEnd Fluorometric TUNEL System, Promega, Madison, WI, USA) according to the manufacturer’s instructions. Nuclei were stained with DAPI.

### 4.12. Annexin V Staining Analysis

Cells (5 × 10^5^) were seeded in 6-cm dishes and incubated for 18 h at 37 °C in a humidified incubator containing 5% CO_2_ in the air. After incubation, cells were treated with DMSO (0.1%) as control vehicle (or control siRNA) and the indicated treatment (or siRNA against IL4Rα or IL13Rα1) for 48 h. Cells were washed with PBS, trypsinized, and resuspended in binding buffer. Cells were analyzed by using a FACSCalibur (BD Biosciences, San Jose, CA, USA), and the data were analyzed by FlowJo (De Novo Software, Glendale, CA, USA). Ten thousand events were collected in each run. The percentage of cells that are undergoing apoptosis was determined by using the FITC Annexin V Apoptosis Detection Kit I (BD PharMingen, San Jose, CA, USA) with propidium iodide (PI, Sigma, St. Louis, MO, USA) according to the manufacturer’s instructions.

### 4.13. Cell Cycle Analysis

Cells (5 × 10^5^) were seeded in 6-cm dishes and incubated for 18 h at 37 °C in a humidified incubator containing 5% CO_2_ in the air. After incubation, cells were treated with DMSO (0.1%) as control vehicle (or control siRNA) and the indicated treatment (or siRNA against IL4Rα or IL13Rα1) for 48 h. Cells were washed with PBS, trypsinized, and fixed in ice-cold 70% ethanol overnight at −20°C. After fixation, the cells were centrifuged at 1350 rpm for 5 min and incubated with a PI solution for 30 min at 37 °C. Cell cycle distribution analysis was performed using flow cytometry (Beckman Coulter, Brea, CA, USA).

### 4.14. Transfection of siRNA and Plasmid DNA

Cells (5 × 10^5^) were seeded in 6-cm dishes and incubated for 18 h at 37 °C in a humidified incubator containing 5% CO_2_ in the air. After incubation, cells were transfected with siRNAs (control (Cat. No.: sc37007), IL4Rα (Cat. No.: sc-35661), IL13Rα1 (Cat. No.: sc-63337), or JAK2 (Cat. No.: sc-39099), 1 nM (Santa Cruz Biotechnology)) or plasmid DNAs (pECE empty/Flag-FOXO3 or pCMV3-C-HA empty/HA-JAK2 plasmid DNA, 1 µg) to 3 µL of Lipofectamine 2000 (Invitrogen) in 300 µL of serum-free media for 6 h at 37 °C in a humidified incubator containing 5% CO_2_ in air. Then cell culture media was replaced with the fresh media containing 10% FBS, and cells were incubated for 18 h.

### 4.15. Statistical Analysis

The CCRCCs immunostained for IL4Rα and IL13Rα1 were grouped to negative and positive cases according to their expression with receiver operating characteristic curve analysis [93,97,98]. The cutoff points of IL4Rα and IL13Rα1 immunohistochemical staining were determined at the highest area under the curve to predict cancer-specific death of CCRCC patients. This study evaluated for cancer-specific survival (CSS) and relapse-free survival (RFS) of CCRCC patients through December 2012. The duration for CSS was calculated as the time from the date of diagnosis to the date of death from CCRCC or last contact. The death from CCRCC was considered an event for CCS analysis. The death from other causes or alive of patients at last contact were treated as censored for CSS analysis. The duration for RFS was calculated as the time from the date of diagnosis to the date of relapse or death from CCRCC, or last contact. The relapse of CCRCC or death from CCRCC was an event in the RFS analysis. The patients who died from other causes or who were alive without relapse at last contact were treated as censored for RFS analysis. The prognostic significance of various clinicopathological factors in CCRCC patients was evaluated by univariate and multivariate Cox proportional hazards regression analyses, and Kaplan-Meier survival analysis. The associations between clinicopathologic factors and the expression of IL4Rα and IL13Rα1 were analyzed by Pearson’s chi-square test. Data are expressed as the mean ± standard error (STE) of three independent experiments. Differences between groups were analyzed with one-way ANOVA when the variances were equal. SPSS software (version 20.0, IBM, palo alto, CA, USA) was used throughout. All statistical tests were two-sided, and *p*-values less than 0.05 were considered statistically significant.

## 5. Conclusions

**Figure 6:**
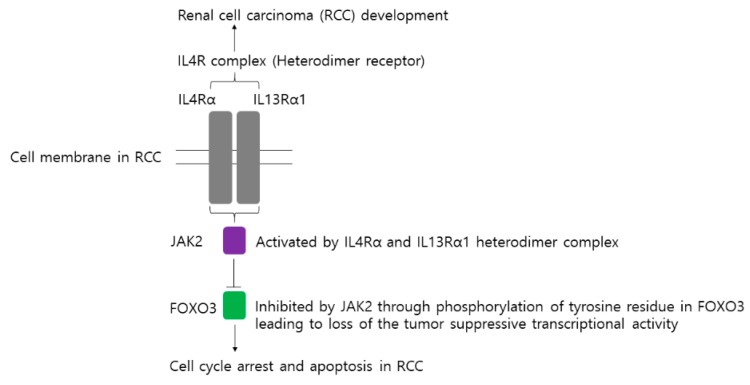
A diagram describes the role of IL4R complex (a heterodimeric complex of IL4Rα and IL13Rα1) in promoting RCC tumorigenesis via phosphorylation of tyrosine residue in FOXO3 by activation of JAK2 leading to loss of the tumor-suppressive transcriptional activity.

Collectively, we investigated a biochemical mechanism to explain the relationship between IL4Rα and IL13Rα1 expressions, as well as the shorter survival see in CCRCC patients [99]. We demonstrated that IL4Rα and IL13Rα1 are associated with the proliferation of RCC cells and the protein stability of FOXO3 via JAK2. We showed a novel signaling pathway underlying the ubiquitination mediated degradation of FOXO3 protein depending on its tyrosine phosphorylation by JAK2, leading to contribute to RCC development. To the best of our knowledge, this is the first report that JAK2 is shown to regulate FOXO3 protein stability. Based on our findings, we proposed a schematic model for a novel role of JAK2 under IL4Rα and IL13Rα1 in regulating FOXO3 protein degradation and promoting RCC tumorigenesis (Figure 6). Our findings imply that IL4Rα and IL13Rα1 as a new prognostic marker. Further, the study implies that JAK2/FOXO3 are new therapeutic targets against RCC. Our findings support the notion that a unique oncogenic JAK2 mediates the degradation of FOXO3 protein and contributes to an understanding of the control of FOXO3 protein in the tumor. 

## Figures and Tables

**Table 1:** Clinicopathologic variables and the expression of interleukin4 receptor α (IL4Rα) and interleukin13 receptor α1 (IL13Rα1) in clear cell renal cell carcinoma (CCRCC) patients.

Characteristics		No.	IL4Rα	*p*	IL13Rα	*p*	IL4Rα/IL13Rα1 Pattern		*p*
			Positive		Positive		−/−, +/−, −/+	+/+	
Sex	Male	140	67 (48%)	0.251	56 (40%)	0.206	101 (72%)	39 (28%)	0.393
	Female	59	23 (39%)		18 (31%)		46 (78%)	13 (22%)	
Age, y	≤55	81	30 (37%)	0.054	27 (33%)	0.352	68 (84%)	13 (16%)	0.007
	>55	118	60 (51%)		47 (40%)		79 (67%)	39 (33%)	
Tumor size, cm	≤7	168	73 (43%)	0.242	58 (35%)	0.070	129 (77%)	39 (23%)	0.029
	>7	31	17 (55%)		16 (52%)		18 (58%)	13 (42%)	
TNM stage	I	162	70 (43%)	0.232	54 (33%)	0.019	125 (77%)	37 (23%)	0.027
	II–IV	37	20 (54%)		20 (54%)		22 (59%)	15 (41%)	
LN metastasis	Absence	197	88 (45%)	0.118	72 (37%)	0.065	147 (75%)	50 (25%)	0.017
	Presence	2	2 (100%)		2 (100%)		0 (0%)	2 (100%)	
Nuclear grade	1	36	18 (50%)	0.055	10 (28%)	0.108	29 (81%)	7 (19%)	0.053
	2	121	47 (39%)		43 (36%)		93 (77%)	28 (23%)	
	3 and 4	42	25 (60%)		21 (50%)		25 (60%)	17 (40%)	
Necrosis	Absence	172	76 (44%)	0.457	61 (35%)	0.205	131 (76%)	41 (24%)	0.063
	Presence	27	14 (52%)		13 (48%)		16 (59%)	11 (41%)	
IL13Rα1	Negative	125	38 (30%)	<0.001					
	Positive	74	52 (70%)						

**Table 2:** Univariate Cox regression analysis of cancer-specific survival (CSS) and relapse-free survival (RFS) in clear cell renal cell carcinoma (CCRCC) patients.

Characteristics	No.	CSS			RFS		
		HR	95% CI	*p*	HR	95% CI	*p*
Sex, male (vs. female)	140	0.281	0.085–0.930	0.038	0.337	0.131–0.864	0.023
Age, year, >55 (vs. ≤55)	118	3.603	1.368–9.486	0.009	2.275	1.104–4.688	0.026
Tumor size, >7 cm (vs. ≤7 cm)	31	3.916	1.833–8.366	<0.001	4.755	2.495–9.065	<0.001
TNM stage, I (vs. II–IV)	37	4.044	1.922–8.509	<0.001	5.354	2.831–10.124	<0.001
LN metastasis, presence (vs. absence)	2	0.049	0–1.4 × 10^6^	0.049	15.801	1.956–127.649	0.010
Nuclear grade, 1	36		1	0.095		1	0.012
2	121	1.223	0.352–4.257	0.751	1.296	0.441–3.808	0.638
3 and 4	42	2.761	0.759–10.047	0.123	3.337	1.097–10.148	0.034
Necrosis, presence (vs. absence)	27	2.502	1.062–5.894	0.036	1.598	0.703–3.631	0.263
IL4Rα positive (vs. negative)	90	4.560	1.848–11.250	<0.001	2.806	1.413–5.570	0.003
IL13Rα1 positive (vs. negative)	74	2.310	1.076–4.961	0.032	2.260	1.185–4.314	0.013
IL4Rα/IL13Rα1, +/+ (vs. −/−, −/+, or +/−)	52	3.748	1.771–7.933	<0.001	3.467	1.833–6.557	<0.001

HR: hazard ratios, CI: confidence interval, TNM: tumor-node-metastasis.

**Table 3:** Multivariate Cox regression analysis of CSS and RFS in CCRCC patients.

Characteristics	CSS			RFS		
	HR	95% CI	*p*	HR	95% CI	*p*
Model 1 *						
TNM stage, I (vs. I–IV)	3.603	1.706–7.607	<0.001	5.246	2.773–9.925	<0.001
Necrosis, presence (vs. absence)	2.407	1.003–5.777	0.049			
IL4Rα positive (vs. negative)	4.334	1.753–10.713	0.001	2.727	1.372–5.422	0.004
Model 2 **						
TNM stage, I (vs. II–IV)	3.507	1.656–7.423	0.001	4.961	2.617–9.404	<0.001
IL4Rα/IL13Rα1, +/+ (vs. −/−, −/+, or +/−)	3.286	1.542–7.000	0.002	3.158	1.662–6.000	<0.001

* The variables included in the multivariate analysis model 1 were sex, age, tumor stage, histologic nuclear grade, tumor necrosis, and the expression of IL4Rα and IL13Rα1. ** The variables included in the multivariate analysis model 2 were sex, age, tumor stage, histologic nuclear grade, tumor necrosis, and co-expression patterns of IL4Rα and IL13Rα1.

## Supplementary Materials

The following are available online at https://www.mdpi.com/2072-6694/11/9/1394/s1, Figure S1: Higher expression of mRNA of both IL4Rα and IL13Rα1 are associated with shorter survival of clear cell renal cell carcinoma patients, Figure S2: Western blot analysis of IL4Rα expression in RCC cell lines, Figure S3: Anti-cancer effect by transfection of siRNA against JAK2 in A498 and ACHN cells. Figure S4: Western blot and densitometry analysis.
