# Supplementary material for: Interleukin4Rα (IL4Rα) and IL13Rα1 Are Associated with the Progress of Renal Cell Carcinoma through Janus Kinase 2 (JAK2)/Forkhead Box O3 (FOXO3) Pathways
Source: Cancers (Basel). 2019 Sep 18;11(9):1394. doi: 10.3390/cancers11091394 (PMC6770213; doi:10.3390/cancers11091394)
Supplement: Supplementary file 1 [file cancers-11-01394-s001.pdf]

Supplementary Materials: Interleukin4Rα (IL4Rα) and IL13Rα1 Are Associated with the Progress of Renal Cell Carcinoma through Janus Kinase 2 (JAK2)/Forkhead Box O3 (FOXO3) Pathways

Mi-Ae Kang, Jongsung Lee, Sang Hoon Ha, Chang Min Lee, Kyoung Min Kim, Kyu Yun Jang and See-Hyoung Park

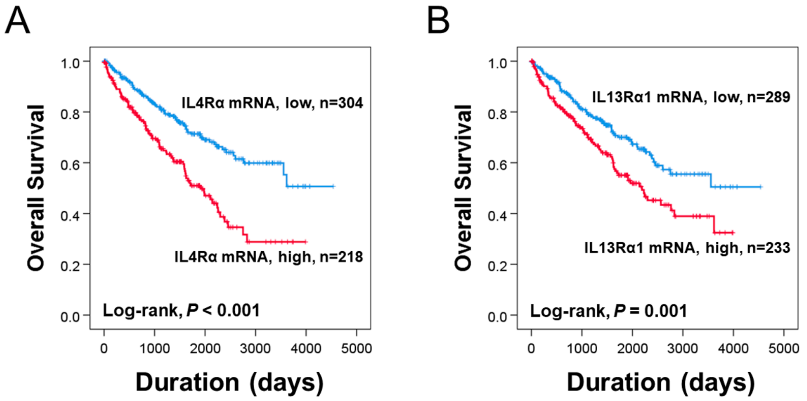

Figure S1. Higher expression of mRNA of both IL4Rα and IL13Rα1 are associated with shorter survior of clear cell renal carcinoma patients. Kaplan-Meier survior analysis according to the RNA expression of IL4Rα(**A**) and IL13Rα1(**B**) in clear cell renal carcinoma. The data for mRNA expression of IL4Rα and IL13Rα1, and survial data of clear cell carcinoma patients were obtained from the OncoLnc database (http:www.oncolnc.org. Accessed 12 July 2019).

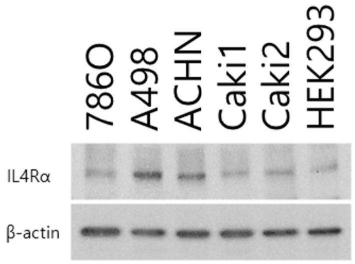

Figure S2. Western blot analysis of IL4Rα expression in RCC cell lines. Β-actin was used for a gel-loading control.

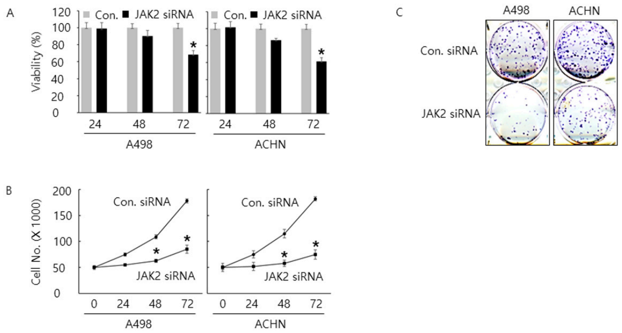

Figure S3. Anti-cancer effect by transfection of siRNA against JAK2 in A498 and ACHN cells. Time-dependent anti-cancer effect by transfection of siRNA against JAK2 in A498 and ACHN cells for 24, 48 and 72 h incubation assay. (**A**) WST-1 assay. (**B**) cell counting assay, respectively. These results are representative data from three biological replicates and the error bar indicates standard error (STE). *indicates the *p*-value < 0.05. (**C**) Anti- colony formation ability by transfection of siRNA against JAK2 in A498 and ACHN cells was deermined by colony formation assay for 14 days after transfection. These results are representative data from three biological replicates.

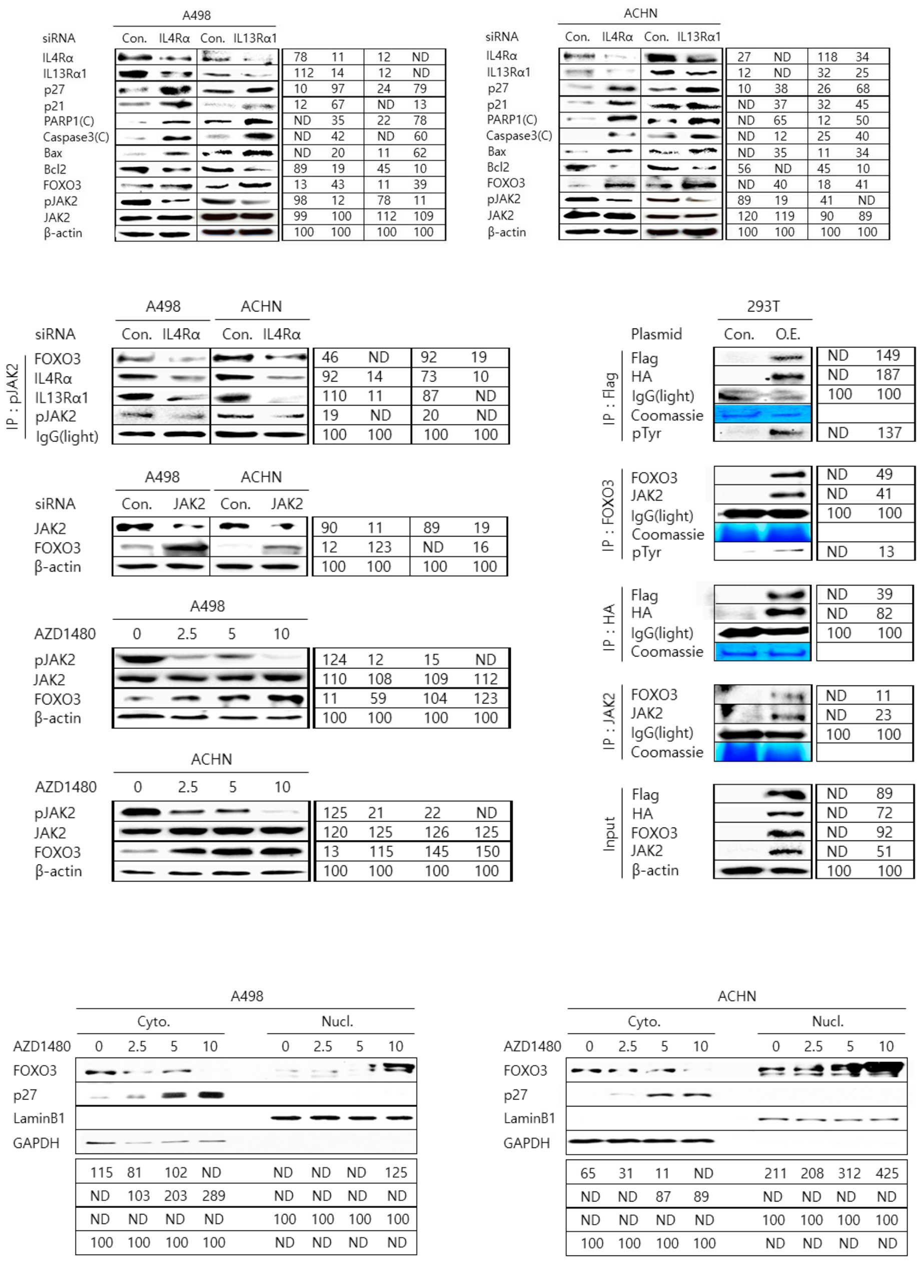
A498
siRNA Con. IL4Rα Con. IL13Rα1
IL4Rα 78 11 12 ND
IL13Rα1 112 14 12 ND
p27 10 97 24 79
p21 12 67 ND 13
PARP1(C) ND 35 22 78
Caspase3(C) ND 42 ND 60
Bax ND 20 11 62
Bcl2 89 19 45 10
FOXO3 13 43 11 39
pJAK2 98 12 78 11
JAK2 99 100 112 109
β-actin 100 100 100 100
ACHN
siRNA Con. IL4Rα Con. IL13Rα1
IL4Rα 27 ND 118 34
IL13Rα1 12 ND 32 25
p27 10 38 26 68
p21 ND 37 32 45
PARP1(C) ND 65 12 50
Caspase3(C) ND 12 25 40
Bax ND 35 11 34
Bcl2 56 ND 45 10
FOXO3 ND 40 18 41
pJAK2 89 19 41 ND
JAK2 120 119 90 89
β-actin 100 100 100 100
A498 ACHN
siRNA Con. IL4Rα Con. IL4Rα
IP : pJAK2
FOXO3 46 ND 92 19
IL4Rα 92 14 73 10
IL13Rα1 110 11 87 ND
pJAK2 19 ND 20 ND
IgG(light) 100 100 100 100
A498 ACHN
siRNA Con. JAK2 Con. JAK2
JAK2 90 11 89 19
FOXO3 12 123 ND 16
β-actin 100 100 100 100
A498
AZD1480 0 2.5 5 10
pJAK2 124 12 15 ND
JAK2 110 108 109 112
FOXO3 11 59 104 123
β-actin 100 100 100 100
ACHN
AZD1480 0 2.5 5 10
pJAK2 125 21 22 ND
JAK2 120 125 126 125
FOXO3 13 115 145 150
β-actin 100 100 100 100
293T
Plasmid Con. O.E.
IP : Flag
Flag ND 149
HA ND 187
IgG(light) 100 100
Coomassie
pTyr ND 137
IP : FOXO3
FOXO3 ND 49
JAK2 ND 41
IgG(light) 100 100
Coomassie
pTyr ND 13
IP : HA
Flag ND 39
HA ND 82
IgG(light) 100 100
Coomassie
IP : JAK2
FOXO3 ND 11
JAK2 ND 23
IgG(light) 100 100
Coomassie
Input
Flag ND 89
HA ND 72
FOXO3 ND 92
JAK2 ND 51
β-actin 100 100
A498
Cyto. Nucl.
AZD1480 0 2.5 5 10 0 2.5 5 10
FOXO3
p27
LaminB1
GAPDH
115 81 102 ND ND ND ND 125
ND 103 203 289 ND ND ND ND
ND ND ND ND 100 100 100 100
100 100 100 100 ND ND ND ND
ACHN
Cyto. Nucl.
AZD1480 0 2.5 5 10 0 2.5 5 10
FOXO3
p27
LaminB1
GAPDH
65 31 11 ND 211 208 312 425
ND ND 87 89 ND ND ND ND
ND ND ND ND 100 100 100 100
100 100 100 100 ND ND ND ND

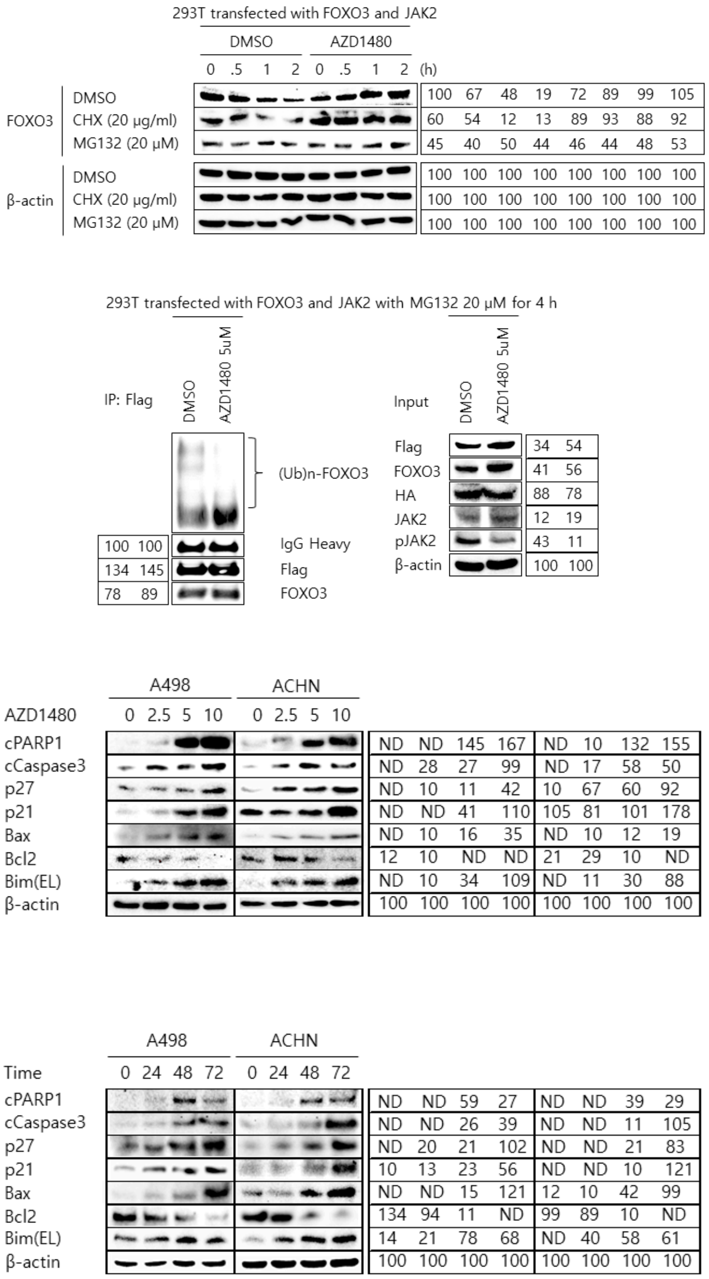

Figure S4. Western blot densitometry analysis. Data are shown as the relative density of each band compared to that of β-actin, IgG, GAPDH, or LaminB1.
